# Enhancing analysis of neo-formed contaminants in two relevant food global commodities: Coffee and cocoa

**DOI:** 10.1016/j.heliyon.2024.e31506

**Published:** 2024-05-17

**Authors:** María E. Medina-Orjuela, Yeison F. Barrios-Rodríguez, Carlos Carranza, Claudia Amorocho-Cruz, Piergiorgio Gentile, Joel Girón-Hernández

**Affiliations:** a Centro Surcolombiano de Investigación en Café (CESURCAFÉ), Universidad Surcolombiana, Av. Pastrana Borrero Carera 1, 410001, Neiva, Colombia; b i-Food, Instituto Universitario de Ingeniería de Alimentos-FoodUPV, Universitat Politècnica de València, Camino de Vera s/n, 46021, Valencia, Spain; c Escuela de ciencias agrícolas, pecuarias y del medio ambiente, Universidad Nacional Abierta a Distancia, Calle 14 Sur # 14 - 23, 111511, Bogotá, Colombia; d School of Engineering, Newcastle University, NE1 7RU Newcastle upon Tyne, United Kingdom; e Department of Applied Sciences, Faculty of Health and Life Sciences, Northumbria University, NE1 8ST Newcastle upon Tyne, United Kingdom

**Keywords:** Neo-formed contaminants, Analytical determination, Acrylamide, Furan, Furfuryl alcohol, Hydroxymethylfurfural, Performance parameters

## Abstract

Neo-formed contaminants (NFCs) are common in many foods, especially those subjected to high-temperature processing. Among these contaminants, products arising from the Maillard reaction, sugar reduction, thermal degradation of polyphenols and lipid oxidation, including acrylamide, furan, furfuryl alcohol, and hydroxymethylfurfural, are consistently linked to potential neoplastic effects. NFCs are found in globally traded commodities like coffee and cocoa, posing a significant risk due to their frequent consumption by consumers. A direct correlation exists between consumption frequency, exposure levels, and health risks. Hence, it's crucial to establish reliable methods to determine levels in both matrices, aiming to mitigate their formation and minimise risks to consumers. This review offers a comprehensive examination, discussion, and identification of emerging trends and opportunities to enhance existing methodologies for extracting and quantifying NFCs in coffee and cocoa. By presenting an in-depth analysis of performance parameters, we aim to guide the selection of optimal extraction techniques for quantifying individual NFCs. Based on the reviewed data, headspace extraction is recommended for furan, while solid and dispersive solid phase extractions are preferred for acrylamide when quantified using gas and liquid chromatography, respectively. However, it is worth noting that the reported linearity tests for certain methods did not confirm the absence of matrix effects unless developed through standard addition, leading to uncertainties in the reported values. There is a need for further research to verify method parameters, especially for determining NFCs like furfuryl alcohol. Additionally, optimising extraction and separation methods is essential to ensure complete compound depletion from samples. Ideally, developed methods should offer comprehensive NFC determination, reduce analysis time and solvent use, and adhere to validation parameters. This review discusses current methods for extracting and quantifying NFCs in coffee and cocoa, highlighting emerging trends and emphasising the need to improve existing techniques, especially for compounds like furfuryl alcohol.

## 1 Introduction

Some compounds that pose a risk to human health, known as neoformed compounds (NFCs) or process contaminants, such as acrylamide (AA), furfuryl alcohol (FA), hydroxymethylfurfural (HMF), and furan, are produced during the thermal processing of some products (>120 °C), such as bread, potatoes, coffee, and cocoa.

The Maillard reaction is the main pathway for AA formation. This is a reaction between an amino group and a carbonyl group derived from amino acids such as asparagine and reducing sugars, respectively [1]. FA is formed by pyrolysis of reducing sugars and thermal degradation of some polyphenols such as quinic acid [2]. On the other hand, caramelisation of sugars is the main mechanism by which HMF is formed. At the same time, furans originate from the breakdown of amino acids and carbohydrates and the oxidation of polyunsaturated fatty acids [3]. These compounds have gained significant attention due to their potential to cause neoplastic and peripheral neuropathic effects [[4], [5], [6]].

Coffee is one of the main sources of exposure to AA, Furan, FFA, and HMF worldwide [7]. Cocoa and its derivatives are consumed at high frequency, which is directly related to exposure to NFCs [8,9]. The AA is found in roasted coffee from 99.8 to 558.8 μg kg^−1^ and in roasted cocoa from 1.1 to 3.2 mg kg^−1^ [3,10,11]. FA concentrations of 223 μg g^−1^ and 321 μg mL^-l^ in roasted beans and in drinks, respectively [12,13]. Occurrence from 300 to 1900 mg kg^−1^ HMF in foods such as coffee [14]. Furans ranging from 4300 μg kg^−1^ to 7400 μg Kg^−1^ and 1 ng g^−1^ have been found in roasted coffee and chocolate bars [15]. Variations in concentrations are influenced by the degree of roasting or, in the case of coffee, the type of species. Robusta species have higher asparagine concentrations than Arabica, a limiting reagent for acrylamide formation [16]. The geographical origin and hydroclimatic conditions of the crop can also influence the composition of NFCs precursors, affecting their formation.

Health and risk control bodies have developed guidelines to minimise NFCs exposure. Since 2017, the European Food Safety Authority (EFSA) has set benchmark food AA levels. Specifically, 400 μg kg^−1^ for roast coffee and 850 μg kg^−1^ for instant soluble coffee [17]. Similarly, since the mid-80s, the California Office of Environmental Health Hazard Assessment (OEHHA) has mandated warning labels for toxic substances such as acrylamide in food [18]. Meanwhile, in 2001 the Joint Food and Agriculture Organization of the United Nations (FAO)/World Health Organization (WHO) Expert Committee on Food Additives (JECFA) agreed on an acceptable daily intake of FA at 0.5 mg kg^−1^ per body weight [2]. On the other hand, although no limit has been set for HMF in roasted coffee and cocoa, the Codex Alimentarius has specified a maximum in processed honey since 2001, not exceeding 40 mg kg^−1^ [19]. Literature provides more information on NFC formation pathways, concentrations, and recommended limits [7].

A crucial step in the NFCs regulation in food is the availability of sufficiently reliable analytical methods for their detection. Although there are several reported protocols for measuring NFCs in food, not all methodologies are appropriate due to the lack of validation processes to support them. The EFSA and the Food and Drug Administration (FDA) have recommended liquid and gas chromatography coupled to single mass analysers (MS) or tandem mass analysers (MS/MS) for determining acrylamide and furan in food [4]. Although these techniques are sufficiently reliable for determining NFCs, their performance in terms of validations performance or merit figures must be reported to ensure suitability. These parameters demonstrate that an implemented method is suitable for its intended use; however, in some studies, this information ought to have been fully reported, leading to decreased confidence in the reliability of the analytical determination.

Furthermore, the nature of the sample significantly influences NFCs analysis methods, critically impacting the extraction, recovery, and quantification of the targeted compound. Hence, choosing a fitting method to detect NFCs in food requires ample information spanning extraction to quantification validation. For this review, articles were consulted articles in the Web of Science and Scopus databases, using "cocoa" and "coffee" combined with each NFC (e.g., acrylamide, hydroxymethylfurfural, furfuryl alcohol, and furan). Then, filtering the title, keywords, and abstract yielded over 40 papers that reported quantification methodologies for NFCs in roasted cocoa and coffee beans and their brews. NFCs in roasted cocoa and coffee beans.

In this review, we critically analyse the advantages and limitations of the currently used extraction methodologies for the most prevalent NFCs (AA, furan, FA, and HMF) found in roasted cocoa and coffee beans and beverages. The effect of the matrix on the appropriate selection of reagents is highlighted. Additionally, quantification methods are scrutinised and detailed, pinpointing validation parameters that signify the reliability of a method. Lastly, we assess novel validation approaches for NFCs determination in roasted cocoa and coffee. These offer more accurate insights for the scientific community concerned with food safety and NFCs traceability.

## 2 Extraction techniques of neo-formed compounds present in coffee and cocoa

**Fig. 1:**
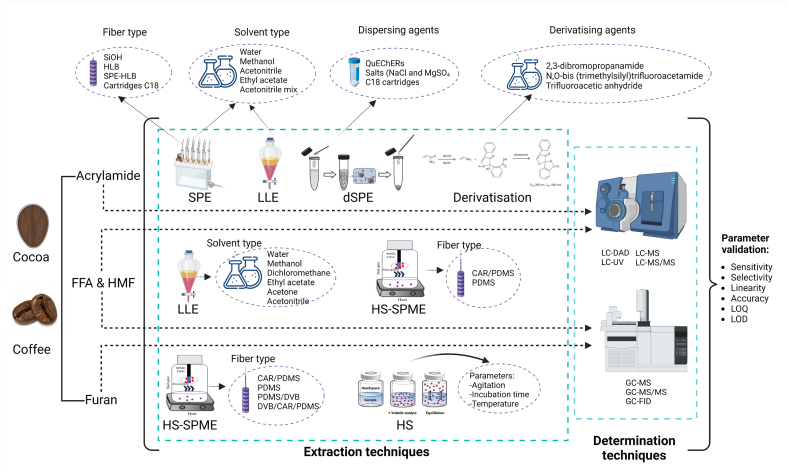
Extraction methodologies applied for the analysis of NFCs in roasted cocoa and coffee samples.

Extraction isolates and concentrates analytes from the matrix, removing potential interferences that may impact subsequent analyses. The extraction techniques vary for each type of NFCs and depend on the employed detection technique (Fig. 1). These procedures typically involve organic solvents such as methanol, ethanol, or acetonitrile; sometimes, formic acid, acetic acid, or water could be added to boost extraction. Conventional and modern techniques such as Ultrasound-Assisted extraction (UAE), Microwave-Assisted extraction (MAE), liquid-liquid extraction (LLE), liquid-phase microextraction (LPME), Single-Drop microextraction (SDME), Hollow-Fibre liquid-phase microextraction (HF-LPME), Dispersive liquid-liquid microextraction (DLLME), Enzyme-Assisted extraction (EAE), Dispersive solid-phase extraction (DSPE) like Magnetic solid-phase extraction (MSPE) or Micro-solid-phase extraction (μSPE) and electric fields speed up the process or enhance mass transfer to the solvent [20].

Following NFCs extraction and quantification, validating method uncertainty is advised, involving performance parameter calculations such as intermediate precision and repeatability, which are expressed as relative standard deviation (%RSD), ideally <15 % for maximum result accuracy. Also, the recovery, which should be 80–110 % [75]. Table 1 displays results reported for these parameters in NFCs; however, some information is inconclusive or non-existent in certain cases. The following section outlines prevalent extraction methods for AA, furan, FA, and HMF in cocoa and coffee matrices, discussing results and performance parameters.

**Table 1:** Validation data settings for analytical methodologies used to determine NFCs in roasted cocoa and coffee.

NFCs	Food matrix	Separation method/mode	Extraction method	IP (% RSD)	Re (%RSD)	Recovery (%)	Lineality (μg Kg-1)	LOD and LOQ (μg Kg-1)	Reference
AA	Coffee	HPLC-MS/MS Column: C18	dSPE-SPE	2.4–6	N.I.	92.00	8-3600 R2: 0.995	N.I.	[53]
		HPLC-MS/MS Column: C18	SPE	N.I.	1.7	99	25-1000 R2: 0.997	LOD: 10 LOQ: 20	[21]
		GC-FID Column: PEG	SPE	N.I.	N.I.	N.I.	N.I.	N.I.	[63]
		HPLC-MS/MS Column: C18	SPE	N.I.	N.I.	83.2	N.I.	LOD: 5 LOQ: 15	[34]
		UPLC-MS/MS Column: C18	SPE	N.I.	N.I.	N.I.	N.I.	N.I.	[27]
		UHPLC-MS/MS Column: PMA	SPE	N.I.	N.I.	N.I.	N.I.	N.I.	[37]
		HPLC-MS/MS Column: C18	SPE	N.I.	N.I.	N.I.	1–100	N.I.	[54]
		HPLC-MS/MS Column: CN	LLE	N.I.	9.2	N.I.	N.I.	LOQ: 30	[62]
		HPLC-MS Column: C18	SPE	N.I.	0.5–1	99–100	1-1000 R2: 0.999	LOD: 2 LOQ: 10	[29]
		GC-MS Column: CP MS	SPE	N.I.	6.3	97.4–108.4	0-300 R2: 0.997	LOD: 10 LOQ: 20.5	[36]
		UHPLC-MS/MS Column: C18	SPE	N.I.	2.8–3.6	94.6–115	100-1000 R2: 0.99	LOD: 5 LOQ: 10	[57]
	Coffee and cocoa	HPLC-MS/MS Column: PGC	SPE	0.6–2.5	9.00	102.9	98-25000 R2: 0.99998	N.I.	[26]
		HPLC-MS/MS Column: PMA	SPE	5.4–15.8	7.7–22.8	N.I.	10–2500	LOD: 9.2 LOQ: 12.5	[31]
	Cocoa	HPLC-MS/MS Column: CN	LLE	N.I.	5–9	84–91	N.I.	LOQ: 30	[62]
		HPLC-DAD Column: C18	SPE	N.I.	N.I.	97.00	R2: 0.998	LOD: 30 LOQ: 100	[9]
		GC-MS Column: 5MS	SPE	N.I.	5.50	98.2	10-5500 R2: 0.9995	LOD: 2.8 LOQ: 8.4	[22]
		GC-MS/MS Column: 5MS	SPE	N.I.	N.I.	N.I.	N.I.	N.I.	[30]
Furan	Coffee	GC-MS Column: CP MS	HS-SPME	N.I.	N.I.	N.I.	N.I.	N.I.	[42]
		GC-MS Column: DVB	HS	6.3	8.4	95.6	2-400 R2: 1	LOD: 1.5 LOQ: 5	[4]
		GC-MS Column: PS-DVB	HS-SPME	11.15–13.25	2.01–9.48	98.31–100.43	0-1000 R2: 0.9992	LOD: 0.11 LOQ: 0.33	[64]
		GC-MS Column: PEG	HS-SPME	1.7–7.1	6.2–13.8	76–101	0-9600 R2: 0.992	LOD: 3 LOQ: 10	[46]
		GC-MS Column: PS-DVB	HS	N.I.	N.I.	N.I.	0–750	N.I.	[39]
HMF	Coffee	HPLC-DAD Column: C8	LLE	0.81–3.3	0.12–0.97	89.94	5000-25000 R2: 0.9997	LOD: 0.11 LOQ: 0.35	[47]
		HPLC Column: C18	dSPE-SPE	N.I.	N.I.	97.2	N.I.	LOQ: 20000	[58]
		RMN	LLE	N.I.	6.9–8.3	101–102	7500–75000	LOD: 6300 LOQ: 22900	[50]
		HPLC-UV Column: C18	HS-SPME	14.23–1.16	0.53–11.98	97.07–106.43	0–500000	LOD: 0.09 LOQ: 0.29	[64]
FFA	Coffee	HPLC-DAD Column: C8	LLE	0.71–3.33	0.68–4.21	91	5000-25000 R2: 0.9982	LOD: 0.76 LOQ: 2.55	[47]
		GC-MS Column: PM	HS-SPME	7.47	3.24	98	1800–99970 R2: 0.9992	LOD: 590 LOQ: 1800	[48]

C18: octyldecylsilane, PEG: polyethylene glycol, PMA: Polymethacrylate, CN: Cyrano, CP MS: (cyanopropyl-phenyl)-methylpolysiloxane, PCG: Porous graphitic carbon, 5MS: (5%-phenyl)-methylpolysiloxane, PS DVB: polystyrene-divinylbenzene, PM: 5 % phenyl-methyl silicone, and N.I.: No information.

### 2.1 Acrylamide extraction

Various methods have been developed for extracting AA from coffee and cocoa matrices. These use the solid phase extraction (SPE), LLE, and dispersive solid phase extraction (dSPE) [21]. Moreover, derivatisation is another technique used for sample preparation in liquid chromatography, involving modifying analyte structures through reactions such as bromination, alkylation, or silylation. These reactions reduce compound polarity and increase volatility, aiding separation. Common derivatising agents for AA include 2,3-dibromopropanamide, N,O-bis (trimethylsilyl)trifluoroacetamide, and trifluoroacetic anhydride [22]. However, this technique has drawbacks, such as the presence of by-products from other matrix compounds or co-extracts that interfere, thereby posing a challenge to the identification and quantification of the target analyte. Consequently, certain extraction procedures can utilise fibres aids, such as solid absorbent or adsorbent fibres, these fibres are typically coated with metal or silica and possess an affinity for specific analyte groups in the sample phase.

SPE, a widely used technique, employs a fixed bed to trap matrix compounds in a liquid phase, followed by solvent elution [23]. It is commonly used for extracting AA from cocoa-derived products and coffee beverages [16,24,25]. Water has shown superior performance for AA extraction in SPE, surpassing organic solvents like methanol, acetonitrile, ethyl acetate, and mixtures ([26,27]; H.-H. [[28], [29], [30]]), likely due to its effectiveness in handling both polar and non-polar compounds. This supports hydrophilic and hydrophobic interactions, facilitating the elimination of undesired substances. Various commercial materials in SPE have been studied, including silica gel (SiOH) and hydrophilic-lipophilic balanced copolymers (HLB) [29]. Using cartridges with these fillers in a single extraction step yielded consistent recovery of the targeted analyte [31]. Yet, a dual extraction approach enhanced recovery values by combining SPE-HLB with multimode techniques (cationic and anionic) alongside LLE [21]. This could be due to the capacity of the LLE to separate lipidic compounds, precipitate proteins, and high-molecular-weight substances by subjecting the liquid sample to a solvent. Combining LLE and SPE in cocoa and coffee matrices for AA extraction achieves selective interference removal due to the affinity of non-polar matrix compounds with the absorbent and AA with the extractant, yielding heightened peak resolution [31]. For instance, using ethyl acetate, analytes can be extracted. After reagent evaporation, the extract passes through multi-mode cartridges (non-polar C18 and mixed-phase ion exchanger), followed by interference precipitation (proteins and high molecular weight compounds) through Carrez I and Carrez II addition [21,31]. Subsequently, centrifugation or shaking accelerates unwanted compound precipitation.

Some studies suggest that lipids are co-extractable in the preparation, with the polarity difference negatively interfering with the recovery of AA and HMF [32]. Cleaning or clarification is important for methods using chromatographic techniques with mass spectroscopy because of the difference between the concentration of NFCs, which are usually trace amounts, and the concentrations of proteins, fibres, and oils. The sensitivity of the equipment determines the minimum detectable concentration of the analyte in the extract, which requires the preparation of extracts with a high concentration of the target analyte. Adequate cleanup is vital in this step to prevent co-extracts from affecting the lifetime of the column due to saturation. High concentrations of interfering compounds can limit the method's sensitivity to the point of contaminating the mass analysers, leading to incomplete ionisation caused by the co-elution of compounds [33].

**Fig. 2:**
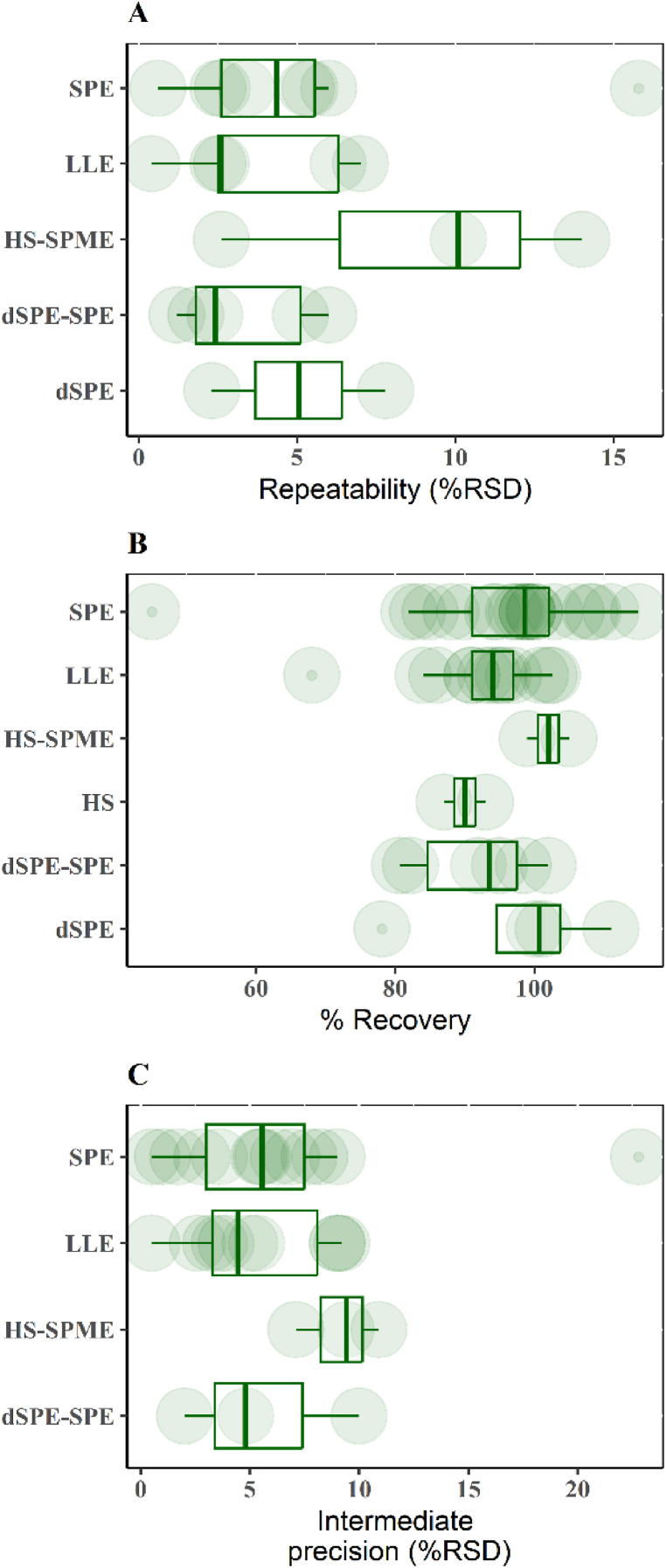
Performance parameters of acrylamide in cocoa and coffee matrices: A) repeatability, B) recovery, and C) intermediate precision.

Dispersive solid phase extraction (dSPE) offers an alternative to traditional SPE. Here, the sorbent is dispersed within the sample matrix, amplifying sorption kinetics and process efficiency. This approach has been effectively utilised for cocoa and coffee samples to eliminate impurities-typically Quick, Easy, Cheap, Effective, Rugged, and Safe (QuEChERs), salts (NaCl and MgSO4), and sorbents (C18 cartridges) [9]. Notably, innovative materials like Fe3O4 nanoparticles, when applied in the QuEChERs technique, have yielded AA recoveries ranging from 81 % to 101 % in coffee [34,35]. Fig. 2(A-C) illustrates the performance parameters of acrylamide in cocoa and coffee matrices: A) repeatability, B) recovery, and C) intermediate precision. When assessing AA quantification methods, reports indicate intermediate precision values between 0 and 15 %, except for an SPE-based study that registered over 20 % [31]. This divergence might stem from Headspace Solid phase microextraction (HS-SPME) fibre selectivity across varying polarity and molecular weight ranges. Recovery data typically fall within 80–120 %, underscoring enhanced AA recovery via SPE and LLE [22,36]. Finally, sample preparation steps might diminish the target analyte concentration, and matrix complexity could result in the co-elution of diverse components (matrix effect). These components, contending for charged surface sites, can compromise method sensitivity and result reliability [37]. To counteract this, employing an internal standard like deuterated acrylamide-3 (AA-d3) through standard dilution is vital for matrix effect reduction [26,31].

Another technique that is rarely used and competes with SPE is DLLME. This consists of extracting the analyte or aqueous sample from fine droplets of the solvent using acetonitrile and dichloromethane as the dispersion and extraction solvents, respectively. The extract was evaporated and reconstituted with 10 % acetonitrile [38]. The recovery ranged from 97 to 106%, indicating high accuracy. Additionally, the intermediate precision and repeatability were consistently equal to or below 9% RSD. This approach aligns closely with the principles of green chemistry that we aim to implement.

### 2.2 Furan extraction and clarification

Furan is commonly analysed through gas chromatography (GC), usually employing deuterated-4 isotope standards. This yields a response factor proportional to the isotope mixture's concentration, described by a linear or exponential equation. This model quantifies the sample analyte and evaluates the matrix effect [39].

Regulatory bodies such as the FDA suggest using HeadSpace (HS), which achieves vapour-sample equilibrium in a sealed vial via controlled temperature and agitation, followed by vial pressurisation and sampling [39]. Adaptations to this technique have been made for cocoa and coffee matrices, mainly adjusting the agitation parameters, incubation time, and temperature. These changes impact vial equilibrium and furan formation due to precursor compounds (sugars and amino acids) in the sample chemical composition. For instance, lowering the incubation temperature from 60 °C to 50 °C reduces furan formation [40]. While the HS technique offers simplicity and time savings over HS-SPME, the latter is more selective, retaining the analyte via adsorption/absorption, and reducing new furan compounds during desorption near 30 °C [41,42]. Aside from agitation, incubation time, and temperature in HS-SPME, the SPME thickness of fibre and material also influence the process. Furan recovery has been compared using various commercial SPME fibres: polydimethylsiloxane (PDMS, 100 μm), carboxene/polydimethylsiloxane (CAR/PDMS, 75 μm), polyacrylate (PA, 85 μm), polydimethylsiloxane/divinylbenzene (PDMS/DVB, 65 μm), carbowax/divinylbenzene (CW/DVB, 65 μm) and a Stable-Flex divinylbenzene/carboxene/polydimethylsiloxane (DVB/CAR/PDMS, 50/30 μm) [43]. CAR/PDMS showed enhanced furan retention due to its affinity for polar and nonpolar molecules through van der Waals forces. Also, 75 μm thick fibres are better for lower boiling point compounds like furan, offering higher selectivity compared to thicker fibres [44]. Notably, a Stable-Flex fibre (DVB/CAR/PDMS, 50/30 μm) was used in prior studies to extract furan from cocoa liquor and chocolate, though specific furan recovery was not reported [45]. The three components of the Stable-Flex fibre are expected to boost the absorption and adsorption of polar and non-polar compounds with lower molecular weights, potentially resulting in greater retention of polar compounds.

The mentioned techniques are widely used for furan quantification, but more information about reliability and accuracy values (intermediate precision, repeatability, and recovery values) is needed. Based on the reviewed data, the methods developed with the HS technique show higher furan recoveries at 95%–105 %, boasting better accuracy (%RSD) < 5.5 % and intermediate repeatability ∼6 % [4]. Conversely, methods with HS-SPME demonstrate precision (%RSD) spanning 1.7 %–13.3 %, with intermediate repeatability ranging from 0.8 % to 13.8 % [46]. Factors such as time and temperature seem to influence vapour pressure and furan solubility, along with salt volume optimisation. The latter loads the furan molecule electrostatically, influencing its mobility towards the coated fibre. Hence, determining an appropriate salt volume involves reducing solvent when the analyte is water-soluble and minimising vapour-liquid equilibrium time [39].

### 2.3 Furfuryl alcohol and hydroxymethylfurfural extraction

LLE serves as the primary technique for extracting FA and HMF (Table 1). The choice of extraction solvent impacts the concentration of the final compound. For instance, when methanol is the solvent, higher recovery percentages of FA and HMF are reported compared to using a water-methanol mixture or pure water; however, this difference is not significant [47]. Furthermore, dipole-dipole interactions and London dispersion forces have been used to extract FA and HMF using solvents of medium to high polarity, like dichloromethane, which solvents of higher polarity such as ethyl acetate, acetone, acetonitrile, and methanol can replace. However, these solvents could introduce interferences, pulling in additional compounds that might contaminate the extract.

To eliminate interferences in coffee and cocoa sample extracts, a common approach involves combining salt addition with filtration or centrifugation, aiding protein precipitation. Also, different fibre types, such as SPME, can be coupled with gas chromatography to isolate FA. In espresso coffee samples, fibres such as PDMS (100 μm), PA (85 μm), and CAR/PDMS (75 μm) have been used [48]. CAR/PDMS fibres have demonstrated heightened FA signal intensities due to electron unpaired charges fostering bonds via van der Waals and dipole-dipole interactions during extraction [48]. Lastly, validation of FA and HMF extraction and quantification through LLE has reported values < 12.5 % RSD for intermediate precision and <15 % RSD for repeatability and recoveries between 80 and 120 % are adequate (Table 1).

## 3 Analytical techniques for NFCs quantification in cacao and roasted coffee

In the last decade, multiple methods have emerged for determining and quantifying NFCs, with chromatographic techniques leading the way. However, alternatives like silver nanoparticle colorimetric methods have also gained attention. This technique relies on a thiol-ene addition reaction between thiol groups in silver nanoparticles and AA in samples. This interaction causes a colour shift from yellow-greenish to grey aggregates [49]. Accordingly, efforts have been made to correlate AA concentration with grey colour intensity through ImageJ. While this could reduce analysis costs and time, the quantification limit (11.1 ng/mL) remains comparable to chromatographic methods.

Other reports have proposed the determination of AA, HMF, and FA by nuclear magnetic resonance (NMR) spectroscopy using pulse length-based connectivity (PULCON), this technique validates specificity, selectivity, sensitivity, and linearity [42,47]. PULCON exhibits sensitivity at concentrations ≥7.5 mg kg^−1^, akin HPLC-UV (≥3 mg kg^−1^), without the need for extra preconcentration or impurity removal steps, reducing analysis time and input volume [50]. While not highly sensitive, it suffices to confirm NFCs presence in coffee beans or brews.

This section will mainly focus on liquid chromatography (LC) and gas chromatography (GC) techniques, as they are the most widely used for separating and quantifying NFCs. Regulatory bodies such as the FDA and EFSA also recommend methods that use these techniques. They rely on compounds in the matrix being drawn to the stationary phase (solid, liquid, or gels) and the mobile phase (liquid or gas), retaining them for a specific period (retention time). Below, we analyse the reported outcomes of these techniques in terms of operational mode, cost, quantification-detection limits, speed, and selectivity for NFCs determination in coffee and cocoa samples.

### 3.1 Liquid chromatography for the determination of AA, FA, and HMF

Liquid Chromatography with diode-array detector (DAD) and ultraviolet and visible light (UV) detectors is often employed for quantifying organic compounds such as AA, FA, and HMF due to their organic nature, water solubility, and hydrogen bond-forming ability [51]. These compounds possess an amide group facilitating hydrogen donation and acceptance, making them detectable within the 210–225 nm wavelength range, dependent on technology. LC-DAD, for instance, can promptly identify AA at a 210 nm wavelength when a slightly acidic polar solvent is utilised. Studies have demonstrated ∼95 % recoveries, an RSD of 3 %, and a Limit of Quantitation (LOQ) of ∼10 ppm, aligning with AOAC recommendations [9]. Water, methanol, acetonitrile, or methanol/water are favoured mobile phases due to their partial charge, varying their ability to dissolve ionic and medium polarity molecules, thereby enhancing separation [52]. Combining these phases with low concentrations (∼1 % v/v) of pH modifiers like acetic acid is common for separation enhancement. Nonetheless, improper pH modifier use can lower signal and intensity [53]. This effect can be attributed to weak hydrogen bonds from acetic acid, heightening solvent affinity for AA and reducing its retention time. Increasing acetic acid concentration (a more polar, non-uniformly charged compound) in the mobile phase diminishes affinity for the stationary phase, shortening elution time for all sample compounds of interest. This results in "shoulders" and tailing peaks, revealing analyte co-elution and chromatographic peak resolution loss.

For stationary phases, the C18 column and reversed-phase ethylene bridge (BHE) hybrid columns are common for AA separation [27,54]. Hybrid columns with high-purity monomers e.g., tetraethoxysilane (TEOS) and bis(triethoxysilyl)ethane (BTEE) enhance stability under high pressures via ethylene bridges, leading to improved chromatogram reproducibility and durability. In contrast, porous graphitised carbon offers heightened overall pH stability, retention, and separation of highly polar molecules like AA compared to C18.

HPLC-MS and HPLC-MS/MS techniques exhibit enhanced sensitivity and selectivity for AA determination in coffee and cocoa. They can detect minute concentrations in coffee samples (>2 μg/L), rendering them ideal for quantifying traces, and they are widely employed [35]. Mass spectrometer analysers gauge the mass-to-charge ratio generated by an electrical or thermal source, crucial for controlling molecule trajectory via electric and magnetic fields [55]. However, some matrices may suppress or overestimation ionisation of the target analyte due to compound competition for charged surface sites, adversely affecting result reliability such as N-Acetyl-ß-alanine, 3-aminopropanamide and lactamide in coffee, and other compounds that can coelute with the analyte. This can be counteracted by employing deuterated isotopes and discussed sample preparation (Section 2), quantifying AA and AA-d3 with a mass-to-charge ratio of *m*/*z* = 72 > 55 and *m*/*z* = 75 > 58 [26]. Electrospray ionisation and atmospheric pressure chemical ionisation are widely used ionisation sources, suitable for a broad molecular weight range and high polarity analytes [26,56,57]. Moreover, organic modifiers such as acetic acid, formic acid, and galli c acid are common in the mobile phase for AA determination. In positive ion mode, these acidify the pH of the solvent to facilitate protonation of the analytes [57].

UV detectors are common in cocoa and coffee FA determination, measuring absorbances at ∼217 nm. Since furan is hydrophilic, C8 or C18 reverse-phase columns can be utilised, with C18 columns having shorter retention times. However, C8 columns provide better peak resolution [47]. Similarly, HMF quantification via LC-DAD and LC-UV at ∼284 nm, using reversed-phase columns, has been reported [58]. Here, the mobile phase often comprises an organic solvent or a higher organic solvent ratio diluted in water, as HMF is hydrophilic and increased aqueous composition reduces elution time. This leads the analyte to possess less affinity for the stationary phase than the mobile phase [59].

Finally, HMF can be quantified through MS/MS using standard addition or an internal standard such as deuterated HMF (HMF-d6), which compensates for the matrix effect. In this case, the sample is typically analysed using an MS/MS spectrometer, tracking charge mass transitions *m*/*z* of 127 and 133 for HMF and HMF-d6 precursor ions, and charge mass ratio of fragment ions 53 and 81 for HMF and 86 for HMF-d6 [60]. An alternative for MS/MS determination is to derivatise HMF with dinitrophenylhydrazine (DNPH), enabling quantification of the HMF-DNPH derivative through selective capillary columns of polymethacrylic acid-*co*-ethylene. This method quantifies ultra-low concentrations, boasting a detection limit of up to 3.4 μg kg^−1^ [32].

### 3.2 Gas chromatography applied to acrylamide and furan determination

Another method for AA quantification involves gas chromatography coupled with mass spectrometry (GC-MS), necessitating prior AA molecule derivatisation. Positive mode electron ionisation detects it. The polarity of the column hinges on the derivatisation type, ranging from medium to low polarity, along with the temperature programme for molecule separation. Commonly, derivatisation technique uses inhibitors e.g., ninhydrin that react with free asparagine, hindering AA formation during thermal desorption. Asparagine is the limiting compound for AA formation in cocoa and coffee samples [61].

To prepare cocoa samples, for GC-MS/MS analysis, bromination-derivatisation with KBr and HBr at acidic pH (1–3) is considered. A stationary phase ratio of phenyl (5 %) with polysiloxane and polyethylene glycol (95 %) is frequently employed to separate acrylamide from 2,3-dibromopropanamide, enabling AA separation from 2,3-dibromopropyramide [30].

Gas chromatography can achieve detection limits comparable to LC-MS/MS for AA quantification. However, it demands more sample preparation steps due to derivatisation, prolonging analytical determination time, increasing uncertainty, error likelihood, and consumable costs, ultimately reducing method efficiency. To address these concerns, direct GC-MS analysis has been suggested, demonstrating a 5 μg kg^−1^ detection limit, recoveries of 80–100 %, and variability <10 % [62]. Gas chromatography with a flame ionisation detector (GC-FID) has also been employed, utilising non-polar fused silica columns and a mixed isothermal programme heating samples to 260 °C, reducing analyte retention time [63]. However, the validation parameters for this methodology have not been reported.

Additionally, due to its volatility, the GC-MS method is well-suited for furan quantification. However, several factors influence this quantification, encompassing sorption temperature, vial equilibrium, and the presence of precursors in cocoa or coffee samples. The GC-MS method mandates sorption temperatures close to 31 °C (furan boiling point), which can be achieved via the HS-SPME approach discussed in section 2. Nonetheless, limited studies have reported recovery data to compare its performance against HS [64]. Conversely, the optimal method for quantifying trace furan levels in cocoa and coffee samples involves integrating a deuterium-labelled standard (d4-furan). This technique necessitates a matrix effect assessment, establishing d-4 furan isotopic addition curves at 50 % above and below the expected concentration to ensure dependable outcomes. Subsequently, it is recommended to validate the hypothesis using a student's t-test at a 95 % significance level [65].

Finally, the stationary phase of the columns is often made of polymers, such as polystyrene-divinylbenzene, phenylpolysiloxane, or cyanopropylphenyl [43,44,66], These form N–H bonds with moderate hydrophobicity or cation exchange mechanisms, facilitating furan retention eluted at ∼230 ± 7 °C [66]. Furthermore, quantifying furan via positive mode electron impact ionisation mass spectrometry establishes *m*/*z* ratios of 68 and 72 for furan and d4-furan, respectively [42; 64].

## 4 Future perspectives and conclusion

The recent advancements in NFCs determination have primarily centred on coffee matrices. Given that compounds such as AA and furan can have comparable concentrations in cocoa, it is crucial to determine NFCs levels in cocoa matrices to minimise potential exposure risks. However, the lipid content of cocoa (>30 %) poses challenges for NFCs extraction and quantification. In this regard, LLE with a dSPE extraction step (salting out) alone is insufficient to eliminate lipid interference, which can retain or prevent the interaction between mildly polar molecules by masking or isolating compounds from the extraction diluent. Employing freezing steps coupled with dispersive techniques for precipitation of higher molecular weight molecules offers an alternative, especially for matrices with higher lipid percentages, in addition with changes in the extract-solvent ratio (>15 %). Furthermore, the occurrence of compounds like lactamide, lactic acid derivatives, and precursors such as N-acetyl-β-alanine in cocoa (formed during fermentation) adds to the complexity. These compounds, prone to fragmentation at source *m*/*z* 72 > 55, could overestimate up to 40 % for NFCs if proper chromatographic cleaning and separation are not attained, manifesting as coeluting peaks or shoulder peaks [37].

Approximately half of the studies concentrated on analysing AA levels. Regarding furan, it is worth noting that HS-SPME demonstrated detection capabilities ten times higher than those of SPE. However, it is important to acknowledge that HS-SPME fibres necessitate nearly twice the extraction time compared to SPE. Conversely, regulatory bodies are yet to establish specific reference techniques for determining FA and HMF in roasted coffee and cocoa. This knowledge gap presents an opportunity for further research in food safety, aiming to devise suitable methodologies for accurate resolution. Such efforts are crucial for formulating effective strategies in the future to mitigate or decrease the presence of these compounds.

Another unexplored field is the development and validation of analytical techniques for quantifying FA. Few studies have addressed this compound in roasted cocoa and coffee. Furthermore, information about verifying method performance parameters and method development, including optimisation of extraction and separation, is limited. These aspects are crucial for showcasing analyte preservation in samples and for confirming the validity of the method through proficiency or interlaboratory tests. This is essential to prevent inaccurate outcomes from extraction shortcomings or sample contamination. Validating methods for assessing FA in cocoa and coffee samples is vital to ensure precise outcomes, assess product quality, establish data comparability and traceability, and facilitate ongoing enhancement of analytical techniques within the cocoa and coffee industry.

The extraction techniques described can be applied to different matrices whose composition or interferences may be similar. However, it is important to know how the matrix under investigation may affect quantification. Simpler methods can be used to avoid loss of analytes during preparation if they do not contain possible co-extracts that may cause matrix effects, as is the case with lipids in cocoa. Concerning extraction methods, the reviewed studies showcased recovery rates exceeding 80 %, as advised by AOAC [75]. Furthermore, the analysed data indicated that the optimal extraction method for furan was dSPE, while SPME and ELL were suggested for AA quantification using GC and LC, respectively. These techniques rely on the selectivity of the materials or consumables employed, achieving satisfactory outcomes through various combinations. However, it is advisable to minimise the number of sample preparation steps to prevent analyte loss. It was impossible to determine which techniques gave the best results for HMF and FA extraction as little information is available for comparison. However, LLE-DAD is suitable for both, according to the information gathered.

Furthermore, emerging techniques such as Solid Phase Magnetic Extraction (MSPE) can be employed to mitigate the matrix effect when quantifying AA. Utilising cysteine-functionalised magnetic adsorbents, which possess modifiers in their covalent organic structure, enhances hydrophilic interaction range and enables heightened selectivity. This selectivity is transferrable not only to carbohydrate-rich matrices but also to lipid matrices, making it adaptable to common analytical methods e.g., LC or GC coupling [67].

Capillary electrophoresis (CE) is a technique that requires only a small amount of sample and has a high separation efficiency and adequate sensitivity. However, additional steps such as derivatisation are required for the analysis of AA [68].

Validation parameters for NFCs determination methods were seldomly reported. High precision and accuracy are vital to ensure confident quantification of NFCs, as results validation plays a critical role in quality control for end products and the authenticity of raw materials in the food industry. Accurate reporting of validation parameters is essential in research studies. While linearity, recovery, and intermediate precision were frequently reported, linearity tests often lacked evidence of matrix effects in methods not developed through standard addition. This leads to uncertainty in reported values. It is imperative to specify the tests used to assess matrix effects, perform statistical analyses, and correct by calibration curves if applicable for reproducibility across laboratories. Regulatory bodies should mandate these measures to reduce uncertainty when measuring analytes such as AA in complex matrices.

Exploring rapid NFCs detection techniques e.g., optical methods is valuable for standardising colorimetric references in control processes. However, precision might need improvement compared to chromatographic techniques. NMR presents another avenue for NFCs assessment. Its streamlined sample preparation, especially with ultrasound-assisted solvent extraction, offers efficiency and time savings. Nonetheless, using solvents and deuterated standards is essential, potentially increasing analysis costs and method complexity.

Synthetic polymer materials with specific recognition sites capable of binding tightly to the target, known as molecularly imprinted polymers (MIPs), coupled to electrochemiluminescent (ECL) sensors, are promising for the determination of AA due to the obtained detection sensitivity of 0.123 nM, the speed of application of the method and the reduction of solvents in the assay [69]. For the comprehensive analysis of NFCs, this method is promising.

Finally, studies have reported the joint determination of NFCs such as acrylamide with HMF or FA [47,[70], [71], [72]]. Due to furan's highly volatile nature, extracting and quantifying it alongside other NFCs is challenging. Currently, no protocol combines the extraction and quantification of acrylamide, FA, and HMF. Nevertheless, compatible extraction techniques like dSPE and SPE can be implemented for these compounds. Optimising the recovery of analytes within the recommended 80–120 % limits is crucial, especially for accurate determination by LC-MS or LC-MS/MS. The cleaning process must minimise interfering compounds while ensuring adequate recovery of target analytes. For instance, using PSA to clean acrylamide may remove aromatic compounds like HMF, making method development a future challenge.

Sample preparation techniques like Stir-Bar Sorptive Extraction (SBSE), which employs PDMS as a sorbent, can determine three NFCs: FA, HMF, and furans [73]. However, it lacks the material variety for enhanced selectivity seen in HS and SPME techniques. SBSE can minimise compound formation during sample preparation due to its room temperature application. Additionally, it significantly reduces solvent use compared to ELL in FA analysis [74]. Exploring chemometrics could aid in comprehensive NFC estimation or prediction. Ultimately, methods for NFC extraction and determination should prioritise sensitivity, speed, and adherence to green chemistry principles.

Acknowledgements.

## Data availability statement

The authors declare that no data associated with our study has been deposited into a publicly available repository since no data was used for the research described in the article.

## CRediT authorship contribution statement

**María E. Medina-Orjuela:** Writing – original draft, Software, Formal analysis. **Yeison F. Barrios-Rodríguez:** Writing – original draft, Methodology, Investigation, Formal analysis, Conceptualisation. **Carlos Carranza:** Resources, Project administration. **Claudia Amorocho-Cruz:** Resources, Project administration. **Piergiorgio Gentile:** Writing – review & editing, Writing – original draft, Methodology. **Joel Girón-Hernández:** Writing – review & editing, Writing – original draft, Funding acquisition, Formal analysis.

## Declaration of competing interest

The authors declare that they have no known competing financial interests or personal relationships that could have appeared to influence the work reported in this paper.
